# Evaluation of portable devices for medicine quality screening: Lessons learnt, recommendations for implementation, and future priorities

**DOI:** 10.1371/journal.pmed.1003747

**Published:** 2021-09-30

**Authors:** Céline Caillet, Serena Vickers, Vayouly Vidhamaly, Kem Boutsamay, Phonepasith Boupha, Stephen Zambrzycki, Nantasit Luangasanatip, Yoel Lubell, Facundo M. Fernández, Paul N. Newton

**Affiliations:** 1 Lao-Oxford-Mahosot Hospital-Wellcome Trust Research Unit, Microbiology Laboratory, Mahosot Hospital, Vientiane, Lao PDR; 2 Centre for Tropical Medicine and Global Health, Nuffield Department of Medicine, University of Oxford, Oxford, United Kingdom; 3 Infectious Diseases Data Observatory (IDDO)/WorldWide Antimalarial Resistance Network (WWARN), University of Oxford, Oxford, United Kingdom; 4 School of Chemistry and Biochemistry, Georgia Institute of Technology, Atlanta, Georgia, United States of America; 5 Mahidol-Oxford Tropical Medicine Research Unit, Faculty of Tropical Medicine, Mahidol University, Bangkok, Thailand

## Abstract

Céline Caillet and co-authors discuss a Collection on use of portable devices for the evaluation of medicine quality and legitimacy.

**Summary points**

- Portable devices able to detect substandard and falsified medicines are vital innovations for enhancing the inspection of medicines in pharmaceutical supply chains and for timely action before they reach patients. Such devices exist, but there has been little to no independent scientific evidence of their accuracy and cost-effectiveness to guide regulatory authorities in choosing appropriate devices for their settings.
- We tested 12 portable devices, evaluated their diagnostic performances and the resources required to use each device in a laboratory.
- We then assessed the utility and usability of the devices in medicine inspectors’ hands in a pharmacy mimicking a real-life Lao pharmacy.
- We then assessed the health and economic benefits of using portable devices compared to not using them in a low- to middle-income setting.
- Here, we discuss the conclusions and practical implications of the multiphase study discussed in this Collection. We discuss the results, highlight the evidence gaps, and provide recommendations on the key aspects to consider in the implementation of portable devices and their main advantages and limitations.

Global concerns over the quality of medicines, especially in low- and middle-income countries (LMICs) are exacerbated by the Coronavirus Disease 2019 (COVID-19) pandemic [1,2]. The World Health Organisation (WHO) estimated that 10.5% of medicines in LMICs may be substandard or falsified (SF) [3]. “Prevention, detection, and response” to SF medical products are strategic priorities of WHO to contribute to effective and efficient regulatory systems [4]. Numerous portable medicine screening devices are available on the market, holding great hope for detection of SF medicines in an efficient and timely manner, and, therefore, might serve as key detection tools to inform prevention and response [5,6]. Screening devices have the potential to rapidly identify suspected SF medical products, giving more objective selection for reference assays, reducing the financial and technical burden. However, little is known regarding how well the existing devices fulfil their functions and how they could be deployed within risk-based postmarketing surveillance (rb-PMS) systems [5–7].

We conducted, during 2016 to 2018, a collaborative multiphase exploratory study aimed at comparing portable screening devices. This paper accompanies 4 papers in this PLOS Collection “A multiphase evaluation of portable screening devices to assess medicines quality for national Medicines Regulatory Authorities.” The first article introduced the multiphase study [8]. In brief, 12 devices (**S1 Table**) were first evaluated in a laboratory setting [9], to select the most field-suitable devices for further evaluation of their utility/usability by Lao medicines inspectors [10]. Cost-effectiveness analysis of their implementation for rb-PMS in Laos was also conducted [11]. The results of these 3 phases were discussed in a multistakeholder meeting in 2018 in Vientiane, Lao PDR (**S1 Text**). The advantages/disadvantages, cost-effectiveness, and optimal use of screening devices in medicine supply chains were discussed to develop policy recommendations for medicines regulatory authorities (MRAs) and other institutions who wish to implement screening technologies. A summary of the main results of the multiphase study is presented in **S2 Table.**

As far as we are aware, this is the first independent investigation comparing the accuracy and practical use from a public health perspective, of a diverse set of portable medicine quality screening devices. The specific objective(s) for which the portable screening technologies are implemented, their advantages/limitations, costs and logistics, and the development of detailed standard operating procedures and training programmes are key points to be carefully addressed when considering selection and deployment of screening technologies within specific rb-PMS systems **(Fig 1)**.

Here, we utilise this research and related literature to discuss the evidence, gaps, and recommendations, complementary to those recently published by the US Pharmacopeial Convention [12]. These discussions can inform policy makers, non-governmental organisations, wholesalers/distributors, and hospital pharmacies considering the implementation of such screening devices. We discuss unanswered research questions that require attention to ensure that the promise these devices hold is realised.

**Fig 1:**
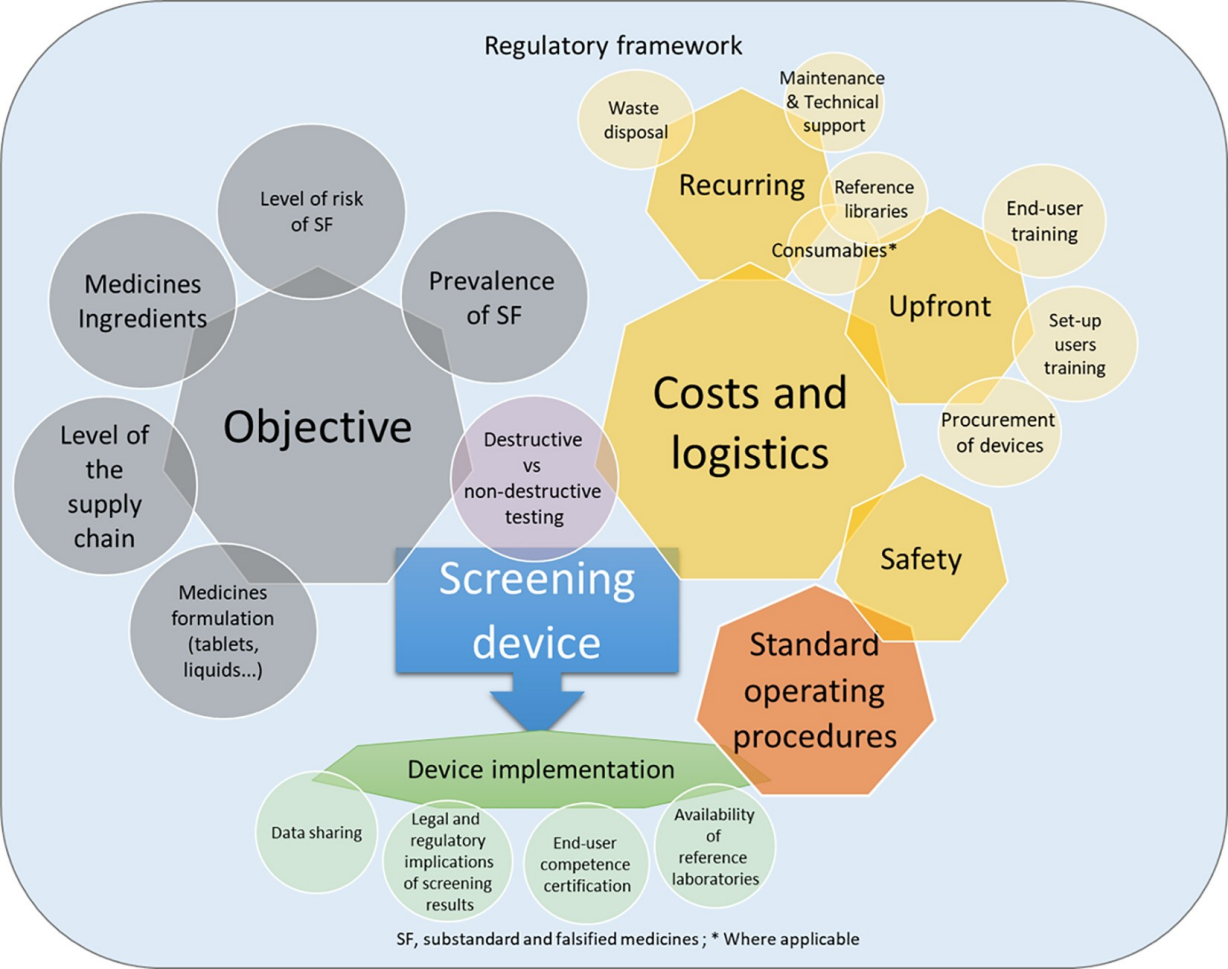
Major proposed considerations for the selection and implementation of medicine quality screening device. Each circle represents a key consideration when purchasing a screening device, grouped by themes (represented by heptagons). When the shapes overlap, the considerations are connected. For example, standard operating procedures are needed for the implementation of devices and should include measures for user safety. The circle diameters are illustrative.

## Regulatory framework

Fast identification of SF medicines will have limited impact in a PMS system if quarantine/recall of suspicious products cannot be acted upon if there is no legal or regulatory recognition of the screening technology results. Relatively few LMICs have access to reference WHO prequalified (or equivalent) medicine analysis laboratories [13]. If they are not available, this will negate the potential benefits of screening devices, as confirmatory testing may not be possible or samples would need to be shipped outside the country, thus increasing costs and time lag. Reference testing is vital to confirm or refute the screening device results and determine the quality defect to allow regulatory risk analysis for patients. It will be important to avoid falsely identifying a good-quality medicine as poor quality that would result in economic and reputational damages to the manufacturer and outlet/facility. Reference analyses will be needed to inform forensic and legal procedures. For countries without WHO prequalified (or equivalent) medicine analysis laboratories, while these are being developed, reference laboratories should be identified to support affordable reference assays. Protocols to maintain the chain of custody of the collected samples should be observed. Country-specific changes to legislation to enable swift and appropriate response to medicines failing screening tests require consideration before the adoption of these technologies.

## The right device for the right objective

The devices we tested have been shown to be generally accurate for the detection of medicines containing zero and/or wrong active pharmaceutical ingredient (API) [5]. Globally falsified and substandard medicines are often sympatric, with variable prevalence through time and space [14,15]. However, none of the handheld devices when used “out of the box” detected with high sensitivity substandard medicines containing incorrect %API. Taking into account the detection abilities of the devices relative to the SF medicines issues in the community in which they are to be used will be key for successful implementation. Introducing devices able to detect falsified medicines, but not substandard medicines into PMS, could result in false confidence in the quality of marketed medicines, particularly where substandard medicines are more widely prevalent. Non-handheld devices with stated abilities to quantify API(s) showed higher sensitivities in our laboratory evaluation for samples containing 50% and 80% of the stated API, but they were not “field-suitable” for pharmacy inspections. If their performances are confirmed on a wider range of medicines, they may be useful screening devices in countries with restricted access to formal high-quality reference laboratories. They could also be considered for use in mobile laboratories, as implemented in Russia [16] or in district/regional offices where samples and chemicals can be more safely manipulated. However, they require experienced technicians and consumables.

Some devices detect the presence/absence of API(s), while others screen the whole medicine formulation, including excipients, such as diluents (e.g., lactose) and lubricants (e.g., magnesium stearate). How advantageous these capabilities are will depend on the question(s) being asked, such as whether a stated API is present or whether a sample stated as brand X made in factory Y, really is brand X made in factory Y. The ability to screen the whole chemical formulation may be advantageous in detecting falsification when criminals add the expected API to fool devices and may be helpful in identifying excipient changes or potentially dangerous adulterants. However, brand-specific testing requires significant resources, as specific methods will need to be developed by brand. Development of methods that would cover all brands of the same API/API combinations are thus preferable. Understanding the typology of local defects found in different SF medicines will help to select the appropriate device(s).

Some devices were limited to testing of 1, 2, or 5 APIs out of the 7 targeted APIs. All the spectrometers included were able to test all medicines containing the 7 APIs tested, although difficulties with ofloxacin samples in the laboratory evaluation were found with 1 near-infrared (NIR) spectrometer. In a review of the scientific literature until April 2018, the median (range) number of APIs assessed per device was only 2 (1 to 20), a small proportion of the approximately 7,000 global international nonproprietary names of pharmaceutical substances [17]. Devices that can only detect one of the APIs in combination formulations cannot properly characterise the whole sample. Chemical insight suggests a priori that some APIs and/or excipients will be problematic for certain devices. For example, Raman scattering from medicines with relatively low concentration of API(s) may be insufficient to yield an API signature, if one of the APIs in coformulated APIs does not provide a unique or strong enough signal [18] and some fluorescent compounds may interfere with key Raman signals. Careful consideration of the abilities of the devices to test compounds (APIs and excipients) are thus crucial. Preimplementation pilot studies of the targeted products are required.

In our study, artesunate powder vials contained only 60 mg API. Such a limited amount made testing with the TruScan RM difficult. It is uncertain whether other API powders in glass vials would raise similar issues. **S3 Table** summarises insights from the team chemists on the levels of difficulty with the devices evaluated to test medicines formulations other than tablets.

The low-cost single-use technologies tested required sample destruction. All but one spectrometer in our study were able to test tablets through transparent packaging with high sensitivities and specificities to identify SF medicines. Of the 14 brands of medicine included in our field evaluation, 10 were in opaque primary packaging. Certain tablet coatings, such as titanium dioxide, and capsule shells likely provide difficult barriers to spectroscopic examination [19,20], inhibiting API content analysis. In the field, nondestructive techniques may need to become destructive for coated tablets and capsules to directly scrutinise the core content. Difficulties with some spectrometers when scanning through packaging may occur if the material generates its own signal. For example, false positives may be observed if blister pack plastic is degraded, but not the medicine it contains, as the spectral signal emitted by the packaging could be altered and the sample then be falsely identified as poor quality. How different plastics used in blisters and capsules influence NIR and Raman spectra with portable devices does not seem to have raised much attention. Assessment of portable devices using spatially offset Raman spectroscopy (SORS) technology that can scan through opaque containers are interesting innovations to pursue [21,22].

Careful consideration of optimal positioning of the devices within supply chains will need to be tailored for each country as there is great global diversity in national medicine supply systems. While handheld spectrometers might be useful at inspecting drug outlets and border checkpoints, other more cumbersome (benchtop size) technologies may be more usable at border checkpoints, in quality control laboratories, in more central offices, or in mobile laboratories [16]. In this study, some “handheld” spectrometers were still perceived by medicine inspectors as cumbersome, heavy, or not easy to use when “handheld” and thus difficult to use in small drug outlets. This requires further investigation in real-life settings and feedback from users to optimise ergonomic design. Extra space needed to carry out the tests was reported as a drawback by distributors [7]. Single-use devices may be useful at border checkpoints and for health workers working remotely in vertical disease control programmes.

The selection of the optimal device(s) should also take into account the risk of SF medicines though time and space. More investment in national and international systems to develop and share risk-based stratification is needed.

## Costs and logistics

The purchase cost of devices and other upfront costs, such as training, may vary significantly by the number of devices purchased and/or the institution that purchases them. Routine use of highly specific devices should aim to reduce the proportion of good-quality samples unnecessarily sent for confirmatory testing in quality control laboratories and hence yield overall savings.

Recurring costs for software updates, device calibration, maintenance, repair, consumables, waste disposal (of the samples tested, consumables, and, in some cases, device parts), staff time, update of spectrometer reference libraries, training of new users, continuous training/proficiency testing, and the cost for device quality assurance/quality control should be taken into account. Logistics considerations for the procurement of consumables and device maintenance and calibration are also important for sustainability. High costs of maintenance, repair and calibration, and the lack of in-country customer services were quoted as major barriers towards the implementation of spectrometers by regulators [7].

For the spectrometers, reference library creation and updates will incur significant costs. We encountered the problem that ultra-performance liquid chromatography analyses demonstrated that some “reference” samples contained API content outside pharmacopoeial limits, despite being procured from what were thought to be reliable sources. Hence, certified reference standards should be used, adding significant costs, time, and human resources. Because the signal detected by spectrometers depends on the whole sample formulation and physical features, procedures should be established to efficiently and promptly update samples or spectra for reference libraries when any changes are made to the medicine formulation; otherwise, false positive results and confusion will arise.

The required level of training and expertise for initial device setup and of end users, and the cost of initial and recurring trainings, should be identified. The creation of reference libraries for spectrometers requires different levels of expertise. In this study, a moderate to high training level was needed to create spectrometer reference libraries. Some technologies (e.g., the C-Vue and the QDa tested in the laboratory evaluation) require significant levels of expertise for both initial setup and end users (**S2 Table**). In the field evaluation, all inspectors were able to successfully complete inspections with all the devices with the training they received, either rudimentary or intensive. Although sample size was small, no significant association between the training level and the performance of the devices was observed. It is likely that for the devices that require end user result interpretation, continuing education, proficiency testing, and quality control will be necessary to optimise device performance. In their screening technology review programme, the US Pharmacopeial Convention (USP) developed a training timeframe matrix. For the 3 spectrometers reviewed, training requirement was estimated to be 1 to 2 hours for a “basic” user, to 2 weeks for an “advanced” user with no technical experience (**S1 Fig**).

Some device components present safety and shipping hazards such as lithium-ion batteries due to their flammable risk, and lasers because of their potential to cause physical damage to the user or property. Lasers are used directly for sampling in Raman instruments and indirectly in instruments such as Fourier transform infrared (FTIR) spectrometers for mirror calibration. As the Progeny spectrometer tablet holder was not adapted for small tablets, the device had to stand vertically on a table to analyse a tablet placed on top, potentially risking exposure to a class 3b risking laser causing eye damage. Training on safety is thus crucial. Technologies using volatile, flammable, acidic or basic, and/or reactive solvents/reagents present chemical/toxicological hazards. Other costs/logistics aspects such as electricity requirements, country-specific import/export duties, and regulations and sensitivity of the devices to environmental factors such as heat should also be considered.

## Device implementation

In the absence of detailed protocols from the device manufacturer, standard operating procedures need to be developed. For example, guidance for how many tests to perform on the same sample and how to interpret the results when tests are discordant need to be developed to optimise device screening accuracy. In the absence of such manufacturer’s guidelines for most devices, when a sample failed the first test of a sample, we chose to operate a “best of three” system for overall sample classification for the devices in the laboratory and in the field evaluations. More data on intertablet variation in spectra and other device output for SF medicines are needed to inform objective protocols. Increase in time and cost for pharmacy inspection with the devices would be exacerbated by repeated testing, but repeated testing would reduce the risk of a single false negative result giving false reassurance about quality or a single false positive result mandating confirmatory testing.

As noted in the field evaluation, significantly less time was spent in the visual inspection of samples during pharmacy inspection with the devices compared to inspection without the devices. False confidence in devices may cause harm by reducing inspectors’ investment in visual inspection. Visual inspection is useful to detect visibly poor quality medicines, both falsified and substandard [23]. Emphasis on the first level of visual inspection of the medicine and its packaging before using screening technology in the second level is of great importance [24,25].

Maintaining a secure chain of custody ensures that medicines in the field can be traced from the collection site to the reference laboratory. Integrated barcode scanners are useful to record the sample identity and may aid operator selection of reference libraries before analysis to reduce operator error. It is recommended to take photographs/scans, in a standardised format, for devices that require visual inspection of results, such as paper analytical devices. The synergistic combination of screening devices with smartphones containing registration, batch number, and packaging information, and alerts of SF medicines with data transfer to and from WHO, holds great promise. This would require significant human and financial capacity and responsive pharmaceutical industries to develop and maintain up-to-date registration databases with regulators.

## The need for more evidence

The evaluation of medicine quality screening devices in laboratory and in real-life settings is in its infancy. Many of the existing commercial spectrometers were not primarily intended for testing the quality of finished medical products, but for raw material testing. Much more research, chemical, economic, sociological, and operational, is needed to develop or adapt devices towards dedicated abilities to detect SF finished medical products. Gaps identified in a recent review of the literature [5] and this multiphase project regarding the testing capabilities of the devices are described in **Box 1.**

**Box 1. Gaps of evidence in our current understanding of the testing capabilities of the devices**

- Device performance tested on a very limited subset of available APIs, predominantly anti-infectives.
- No unified information source on the chemical composition of all essential medicines and excipients to inform which technologies are likely to detect which APIs/excipients (e.g., list of fluorescent compounds, or which API/excipients have specific spectral features).
- No or limited evidence on testing performances for small molecules, hormones (such as oral contraceptives), vaccines, biologics, or cell-based therapies.
- Limited evidence that field-evaluated devices could accurately quantitate API or perform dissolution testing
- Very limited evidence on the ability of the devices to test through packaging and the type of packaging that is least obstructive to device use.
- Very limited discussion of the inability of Raman or IR spectroscopy to test capsules nondestructively due to the opacity of capsule coating.
- No systematic studies looking at the effect of tablet coatings on device performance.
- Very limited testing by the devices of liquid or parenteral formulations; no data on testing of topical formulations or vaccines.
- No testing or comment on the ability of the devices to selectively distinguish between chiral enantiomers.
- Very limited guidance on how to assess and report the performance of medicine quality screening devices to enable comparison between technologies.
- Paucity of independent comparative evaluation of the majority of devices, particularly in field settings.
- No discussion of the risks of criminals designing falsified medicines to evade detection by devices.

**Box 2** lists the gaps of knowledge for optimised and sustainable implementation of the devices within pharmaceutical supply chains.

**Box 2. Gaps of knowledge for optimised and sustainable implementation of the devices**

- Very limited discussion on where in the pharmaceutical supply chain which devices are best deployed.
- Little comment on training needs for sustainable accurate use of the devices.
- Integration of devices into current inspection procedures (of packaging, drug registration, and expiry date) needs to be carefully considered in order to optimise their utility.
- No evidence on the best sampling policies needed to determine which samples to test and how many tests to perform to reduce the risk of a single false negative or positive result.
- Limited consideration of the pharmaceutical industry role in provision of good-quality specimens with which to construct reference libraries.
- No consideration of external quality assurance system to regulate device accuracy and performance.
- Minimal consideration of safety implications for widespread use of lasers and chemical hazard advice for devices requiring chemical handling.
- No discussion of the risks of generating false confidence in the quality of medicines through using devices without proper visual inspection.
- No consideration of infrastructure changes (increased laboratory capacity; financial cost) necessary to accommodate likely increase in samples requiring confirmatory pharmacopoeial testing.
- Improved accuracy of cost-effectiveness analysis will only be possible with more accurate knowledge of the baseline prevalence of SF medicines and the processes and costs of regulatory inspection in different countries.
- Limited investigation of the transferability of reference spectra and methods between instruments.

Substandard medicines have been found in most recent large surveys in LMICs [26–28], but there are no robust validated methods to perform API quantitation with portable spectrometers. Diverse results depending on the technologies, API, formulation tested, and whether destructive or nondestructive methods were employed were described in recent studies [22,29–31]. Some spectrometers include software such as the Trutools chemometrics package (an add-on for TruScan RM) that may simplify quantitation [21]. Good %API prediction performance with the TruScan RM Trutools, even for the quantitation of different API in some coformulated samples, were obtained by analysing the mean spectra at 3 points on tablets [29]. Significant technical work from chemometricians is required to prepare calibration mixtures and develop accurate quantitative models. Further research and innovation are needed for the development of techniques and devices for user-friendly quantitative screening of %APIs. The development of affordable devices (in terms of time/resources consumed and user skills required) for dissolution testing is in its infancy. Research to investigate devices for quantification of drug release to probe differences in dosage form composition are underway [32,33].

Standardised guidance on how to assess and compare the performance of screening devices both in the laboratory and field settings and standardised guidelines to report results are urgently needed [6]. A recent stimulus article from the USP begins to addresses this [12]. Particular attention to “translation” of results to MRA implementation is crucial.

We observed operator errors in the field risking reduction in the accuracy of devices and wider field evaluation in different environments would aid better understanding of training needs and minimise user errors prior to large-scale deployment.

The spectrometers included stored reference spectra in different file formats, which may not be transferable between devices. Discussions between manufacturers developing similar devices on industry standards for spectral file format and transferability between devices are needed. Engineering all devices so that reference signatures can be created by the user without an external computer, with secure cloud-based storage systems, will be time and resource saving. The sharing of standardised reference spectra between MRAs and with manufacturers, both innovative and generic, will also be vital. Ideally, all reference samples of medicines provided by manufacturers or MRAs should come with updated electronic files, cloud downloadable, in the appropriate format for the devices being used. If pharmaceutical manufacturers or MRAs are able to upload NIR/Raman spectra to secure cloud repositories, to automatically update a country’s screening devices as formulations change or new products become available, there are likely to be significant public health benefits.

Much more coordinated evaluation of these devices is needed for the great diversity of medicines through an independent platform, working with regulators and pharmaceutical companies to evaluate devices using standard protocols and samples. When more information is available on the advantages and limitations of individual devices, exploration of combinations of devices with different applications may show practical synergies.

As research expands on screening devices for testing different APIs, especially those coformulated, care will be needed with the public release of data to avoid facilitating criminals making poor quality medicines to circumvent detection of their “products” by the screening devices. The development of consensus targeted product profiles for devices for different positions in supply chains and SF risks would help inform further discussion and innovation.

## Conclusions

We assessed the performances, usability/utility, and cost-effectiveness of implementation of devices that can assist in the detection of substandard and falsified medicines from different viewpoints. We conclude that, even if current technology does not enable one device to effectively monitor the quality of all medicines, there is great promise for portable devices to empower regulatory authorities in their key function to improve national and global public health. The devices assessed in our work have capabilities useful in specific contexts and needs. However, one should also be aware of their limits and difficulties that may arise for their implementation. With the current state of knowledge, for the devices to fully realise their promise, more than technological advances embedded in these devices will be required to ensure that their use has the intended impact in mitigating the devastating effects of substandard and falsified medicines on global health.

## Supporting information

S1 Table Devices included in the study. Devices in bold were included in both laboratory and field evaluation phases.
(PDF)

S2 Table Summary of the main results per device. (PDF)

S3 Table Degree of difficulty to analyse different medicines formulations with the devices included in the study, relative to the analysis of a tablet. (PDF)

S1 Text Summary of the multistakeholders meeting. (PDF)

S1 Fig Training time frame requirements matrix developed by the US Pharmacopeial Convention for 3 spectrometers. Extract from the USP technology review reports of 3 devices: CBEx (Raman), ASD QualitySpec (NIR), and Target-ID (FTIR) (see https://www.usp.org/global-public-health/technology-review-program). Basic user: a user with the ability to follow a standard operating procedure or work instruction to set up and run the instrument and collect data. Intermediate user: a user with the ability to develop and modify methods and evaluate and interpret results. Advanced user: a user with the ability to train other staff and perform basic troubleshooting. Nontechnical experience: a trainee with no prior laboratory experience and no background in one of the physical sciences (e.g., chemistry). Technical experience: a trainee with prior experience working in a laboratory and/or a background in one of the physical sciences. Specialised experience: a trainee with theoretical and practical experience using the technology or the technique underpinning the technology. FTIR, Fourier transform infrared; NIR, near-infrared.
(PDF)

## Abbreviations

API: active pharmaceutical ingredient
COVID-19: Coronavirus Disease 2019
FTIR: Fourier transform infrared
LMICs: low- and middle-income countries
MRA: Medicines regulatory authority
NIR: near-infrared
rb-PMS: risk-based postmarketing surveillance
SF: substandard or falsified
SORS: spatially offset Raman spectroscopy
WHO: World Health Organisation
