# Supplementary material for: Evaluation of portable devices for medicine quality screening: Lessons learnt, recommendations for implementation, and future priorities
Source: PLoS Med. 2021 Sep 30;18(9):e1003747. doi: 10.1371/journal.pmed.1003747 (PMC8483386; doi:10.1371/journal.pmed.1003747)
Supplement: S1 Table — Devices in bold were included in both laboratory and field evaluation phases. (PDF) [file pmed.1003747.s001.pdf]

S1 Table. Devices included in the study. Devices in bold were included in both laboratory and field evaluation phases

Device name	Manufacturer or Institution	Market status	Technology<br>Main Specification	Handheld	Cost[a,b]
**4500a FTIR Single Reflection**	Agilent Technologies	M	FTIR-MIR<br>Spectral range<br>4000cm^{-1}-650cm^{-1}	N	US$ 31,067
**C-Vue**	C-Vue	M[c]	Liquid chromatography	N	One unit with 214nm detector: ~US$ 4,950<br>Stationary Column: ~US$ 370<br>Additional 254 nm detector: ~US$ 1,295<br>Accessories for sample preparation : ~US$ 175
**Minilab**	Global Pharma Health Fund E.V.	M	TLC, disintegration test	N	US$ 2,510 (without reference standards)
**MicroPHAZIR RX**	ThermoFisher Scientific	M	FTIR - NIR<br>Wavelength range<br>1600nm-2400nm	Y	US$47,500
**Neospectra 2.5 (SWS62221-2.5)**	Si-Ware	M	FTIR-NIR<br>Wavelength range<br>1350nm-2500nm	N	Neospectra 2.5: US$ 3,000<br>Light Source: US$1,030<br>White Reference Tile: US$310<br>Fiberoptic Cable and Probe: US$1,261<br>Probe Holder: US$67.83
**NIR-S-G1**	Young Green Energy –Innospectra[d] (the Global Good Fund developed the smartphone application)	M[e]	NIR - Dispersive<br>Wavelength range<br>900nm-1,700nm	Y	US$1,199 (without smartphone)
**Paper Analytical Device**	University of Notre-Dame and Veripad (Kenya, New-York and Boston)	D	Paper-based colour test	Y (S)	US$3
**PharmaChk**	Boston University	D	Microfluidic device with luminescence detection	N	Unknown[f]
**Progeny**	Rigaku	M	Raman<br>1064 nm laser	Y	(ex-demo model)
**TruScan RM**	ThermoFisher Scientific	M	Raman<br>785 nm laser	Y	US$ 62,500 (including chemometric software package and tablet holder)
**Unnamed-Lateral flow immunoassay**	China Agricultural University of Beijing and University of Pennsylvania	D	Lateral flow immunoassay dipsticks	Y (S)	US$ 2-3[g]
**Single-quadrupole QDa MS**	Waters	M	Mass spectrometry	N	US$ 76,169

D: Under development, FTIR: Fourier Transform Infrared, M: Marketed, MS: Mass spectrometry, N: No, NIR: Near infrared, Y: Yes, HPLC: High Performance Liquid Chromatography, NIR: Near Infrared, MIR: Mid-Infrared, TLC: Thin-layer chromatography, S: Single-use device

a Ordering several devices to the manufacture is subject to potential reduced purchase cost

b The costs reported here do not include VAT and may vary by country of purchase

c The device is available for purchase but has been only used as an educational tool

d At the time of the study the NIR unit was produced by Young Green energy. It has been taken over by InnoSpectra Corporation.

e The near-infrared sampling unit is marketed but the smartphone application is not

f The device was lent by the developer and is still under development, and not available for purchase as far as we are aware

g Cost estimated by the manufacturer. The device is not marketed yet and is subject to variation. Purchasing several RDTs is subject to potential reduced purchase cost.
