# Supplementary material for: Evaluation of portable devices for medicine quality screening: Lessons learnt, recommendations for implementation, and future priorities
Source: PLoS Med. 2021 Sep 30;18(9):e1003747. doi: 10.1371/journal.pmed.1003747 (PMC8483386; doi:10.1371/journal.pmed.1003747)
Supplement: S2 Table — (PDF) [file pmed.1003747.s002.pdf]

S2 Table. Summary of the main results per device. The performances in red font are those consistent with the ability of the device as stated by the manufacturer/developer. Devices in orange boxes were not tested by Lao medicine inspectors.

These summary results must be interpreted with caution and in light of the caveats as discussed in the relevant articles of the series, especially in relation to the small sample size of samples and APIs. The results cannot be generalized to other medicines. In this table, the most proximal point of the pharmaceutical supply chain is the raw materials manufacturer; and the most distal point is the patient.

Device (N° of API tested in the study)	Ability to identify 0% and wrong API content samples (laboratory results)	Ability to identify 50% and 80% API content samples (laboratory results)	Advantages	Limitations	Supply chain location where the device could be useful[a]	Level of training (chemist opinion)	Notes	Suggestions for improvement
**4500a FTIR**<br>**(All seven)**	100%<br>(93.3-100%)	28.6%<br>(15.7-44.6%)	No need to select specific reference library prior to scan<br><br>Identification of the API with matches for medicines of unknown identity<br><br>Straightforward interpretation: few user errors in field evaluation and results trusted by users (table of matches appreciated)<br><br>Shorter total time per sample compared to PADs and Minilab.<br><br>Shorter total time of analysis[b] compared to other spectrometers except MicroPHAZIR RX<br><br>Inspectors found easy to use, with on screen step-by-step protocol	Reference library creation needed<br><br>Destroys sample<br><br>Large number of steps required to perform analysis<br><br>Mistakes in naming of samples tested could affect traceability of inspection<br><br>Longer total testing time per sample than other spectrometers. Longer time spent in pharmacy compared to without device inspection<br><br>Occasional freezing of the software<br><br>Heavy weight<br><br>Computer or smartphone required for sample testing	Manufacturers and distributors sites<br><br>Border checkpoints or in a laboratory setting<br><br>Multiple steps, weight, and need for space limits use in pharmacy outlets	To create the library and software:<br>Technician level training at a bare minimum. Biggest difficulty is setting up the correct folder and software set up. Experiment to collect spectra as simple as questioned samples analysis<br><br>To test a sample:<br>Medium: Follow the on-screen instructions. Operator needs to know some problems that arise from not having enough sample or the sample is not pressed enough. Problems with Mid-IR analysis should be also known	Sampling[c] phase longer due to crushing of samples and cleaning device between samples	To integrate a container to collect waste from crushed samples[a]<br><br>Computer screen could be integrated into the lid of the suitcase in which the device is held[a]<br><br>Algorithm for detected reduced API samples

Device (N° of API tested in the study)	Ability to identify 0% and wrong API content samples (laboratory results)	Ability to identify 50% and 80% API content samples (laboratory results)	Advantages	Limitations	Supply chain location where the device could be useful[a]	Level of training (chemist opinion)	Notes	Suggestions for improvement
**C-Vue (Three)**	100%<br>(82.4-100%)	100%<br>(81.5-100%)	Correct identification of all 50 and 80% API medicines, with quantitation of API<br>Intuitive system for experienced analysts<br>Intuitive software for data collection and analysis	Intensive operation and set-up<br>Two computers required to run dual detector set-up<br>Destroys sample<br>Chemicals required	Capital and provincial laboratories by experienced analysts as alternative to formal HPLC for detecting falsified and substandard medicines<br>High level screening device for MRAs without a reference laboratory	To create the library and software: N/A<br>To test a sample: High: User must be able to prepare chemical solution and understand how to dilute samples (including the mathematics behind it). Must be able to create calibration curves. Ideally understands how a column works and potential troubles with such columns. Must be able to use s spreadsheet like software (Excel) and generate calibration curves and integrate chromatographic peaks.		Adaptation so that only one computer is required for dual detection<br>Simplification of setup

Device (N° of API tested in the study)	Ability to identify 0% and wrong API content samples (laboratory results)	Ability to identify 50% and 80% API content samples (laboratory results)	Advantages	Limitations	Supply chain location where the device could be useful[a]	Level of training (chemist opinion)	Notes	Suggestions for improvement
**MicroPHAZIR RX (All seven)**	100% (92.5-100%)	50% (32.9-67.1%)	Averaging spectra for reference library creation possible to take into account variability between batches or within batches<br><br>Analysis through packaging: good performance through blister plastic and replacement packaging (incl. glass vial)<br><br>Barcode reader to 1/enhance traceability 2/reduce analysis time spent to entering samples details<br><br>Good sensitivity to identify 50% API samples in laboratory evaluation<br><br>Easy to use for end user<br><br>Initial instrument set-up straightforward<br><br>Second fastest test time per sample<br><br>Sample window indicator helpful and providing additional confidence in results<br><br>Does not destroy sample & computer not needed	Reference library creation needed<br><br>Calibration and set-up of the device relatively prolonged<br><br>Need to select reference library prior to analysing - subject to user errors<br><br>Low sensitivity to identify 80% API samples in laboratory evaluation<br><br>Small tablets hard to scan - might reduce the performances due to light interference<br><br>Processing of reference libraries creation and updating not straightforward<br><br>Longer time spent in pharmacy compared to inspection without device<br><br>Heavy weight<br><br>Buttons hard to press	Screening for falsified medicines throughout proximal supply chain	To create the library and software:<br><br>Significant training and assistance needed to set-up computer. High level of training need to create library, specifically in converting the initial signatures (spectra) and developing a method for each medicine when testing.<br><br>To test a sample:<br><br>Medium: Needs to be able to conduct calibration check and understand how to conduct experiments.	Self-corrected user errors (selection of wrong library) has been observed in the field<br><br>Barcode reader could not be tested in this study but its use would likely reduce library selection errors by users<br><br>Device froze once in an Evaluation Pharmacy inspection resulting in the loss of records but this was not mentioned by other inspectors, nor by the investigator team and chemist	Suggestions to improve the pistol grip design convenience[a]<br><br>Touchscreen system[a]<br><br>Algorithm for detecting reduced API samples

Device (N° of API tested in the study)	Ability to identify 0% and wrong API content samples (laboratory results)	Ability to identify 50% and 80% API content samples (laboratory results)	Advantages	Limitations	Supply chain location where the device could be useful[a]	Level of training (chemist opinion)	Notes	Suggestions for improvement
**Minilab**<br><br>**(All seven)**	100%<br><br>(93.3-100%)	59.5%<br><br>(43.3-74.4%)	Electricity not required<br><br>Good sensitivity to identify 50% API samples in laboratory evaluation<br><br>Possibility to run several samples of the same API concurrently<br><br>Step by step protocols well described, illustrated and detailed<br><br>Ability to identify the absence or presence of the API	Destroys sample<br><br>Limited sensitivity to identify 80% API samples in laboratory evaluation<br><br>Longer total time per sample than any other devices<br><br>Large<br><br>Chemicals required<br><br>Safety hazards and waste due to chemicals used<br><br>Difficulties to source and unaffordable costs associated with procurement of reference standards, consumables, and TLC plates	Provincial level facilities with some laboratory infrastructure<br><br>Screening in wholesalers	To create the library and software:<br><br>N/A<br><br>To test a sample:<br><br>Medium/High: User must be trained in the safe handling of the chemicals. Training of TLC and how to best spot and develop plates recommended	All 50% API samples (n=2) wrongly identified as genuines by technicians<br><br>Longest sampling (sample and reference solutions preparation, and TLC run) and analysis times	Hazard guidance statements for chemical safety
**Neospectra 2.5 (All seven)**	100%<br><br>(92.5-100%)	5.6%<br><br>(0.7-18.7%)	Analysis through packaging - good performance through blister plastic and replacement packaging (incl. glass vial)<br><br>Easy to set-up<br><br>Small size	Reference library creation needed<br><br>No ability to computationally compare the spectra in the original software provided - observer dependent<br><br>Computer required<br><br>Limited sensitivity to identify 50% and 80% API samples in laboratory evaluation using visual analysis (except ART and some DHAP 50%API samples)	Manufacturers and distributors sites for detecting falsified medicines	To create the library and software:<br><br>Basic computer knowledge needed for software operation<br><br>To test a sample:<br>Medium/High: Needs to be able to conduct calibration check and understand how to conduct experiments. User spectra		Computational spectral comparisons<br><br>Algorithm for detecting reduced API samples

Device (N° of API tested in the study)	Ability to identify 0% and wrong API content samples (laboratory results)	Ability to identify 50% and 80% API content samples (laboratory results)	Advantages	Limitations	Supply chain location where the device could be useful[a]	Level of training (chemist opinion)	Notes	Suggestions for improvement
						interpretation required		
**NIR-S-G1 (All seven)**	91.5% (79.6-97.6%)	30.6%<br><br>(16.3-48.1%)	Good sensitivity to identify 50% and 80% SMTM samples in laboratory evaluation<br><br>Small and light device<br><br>Easy-to-use for end user (smartphone greatly appreciated)<br><br>Fastest testing time per sample compared to other devices. Shortest time spent in pharmacy compared to other devices (not different than inspection without device)<br><br>Fast analysis<br><br>Computer not needed<br><br>Analysis through packaging[d]: good performance through blister plastic and replacement packaging (incl. glass vial)	Poor sensitivity for simulated OFLO and AZITH 50 and 80% samples in laboratory evaluation<br><br>User errors because of wrong selection of reference library<br><br>Lack of capability to create and update reference library by end users<br><br>Lack of ability to input identification information to the spectra files (sample details), limiting data traceability<br><br>Lack of calibration function and performance quality checks by the user<br><br>Not able to test liquids without pre-treatment[e]<br><br>Its small size and less robust aspect made the NIRScan look less reliable than other devices presented in the multi-stakeholders meeting according to regulators	Screening for falsified medicines throughout proximal supply chain	<u>To create the library and software:</u><br><br>At the time of the study must be done by software developer (developer are investigating other options)<br><br><u>To test a sample:</u><br><br>Low: Must be capable of operating smart phone and apps	Self-corrected user errors (selection of wrong library) has been observed in the field<br><br>Latest version of the device (not evaluated in this study) contains calibration check (with a piece of plastic)	Ability to create reference libraries by end users<br><br>Check other APIs for issues similar to that encountered with OFLO
**PADs**<br><br>**(Five[f])**	100%<br><br>(88.8-100%)	0%<br><br>(0-11.6%)	Easy to use for end user<br><br>No electricity required<br><br>No other chemicals than water required	Results interpretation difficult, requires fair level of training and practice[g]<br><br>Potential cross-contamination of cards if contaminated water used for several tests<br><br>Slower analysis time compared to other devices (except Minilab)	Screening at low level pharmacies for specific APIs<br><br>Remote health workers in pre-existing	<u>To create the library and software:</u><br><br>Low: Manufacturer Developed<br><br><u>To test a sample:</u>	Analysis phase[b] longer than other devices but several samples can be run at the same time	An automated application system for reading cards likely to improve results interpretation

Device (N° of API tested in the study)	Ability to identify 0% and wrong API content samples (laboratory results)	Ability to identify 50% and 80% API content samples (laboratory results)	Advantages	Limitations	Supply chain location where the device could be useful[a]	Level of training (chemist opinion)	Notes	Suggestions for improvement
			Three out of four reduced API samples correctly identified as failing in the field evaluation	Sample destruction/samples preparation<br>Need for space<br>Poor sensitivity to identify 50% and 80% API samples in the laboratory evaluation<br>Short shelf-life<br>Colour blind people and user-dependent reading of colours limiting the interpretation of results<br>Instability under tropical conditions	diseases programs<br>Distal supply chain for screening for samples containing zero API<br>Factories without laboratories to screen raw materials<br>Laboratory, border checkpoints	Low/Medium: The experiments are easy to conduct. The primary difficulty is interpreting the data. Could be further simplified with the smartphone app that was being developed, but was not evaluated in this study.	Medicine inspectors were not confident in their abilities to correctly crush and spread the samples on the PADs	(development ongoing)<br>Expansion for more APIs<br>More standardized preparation and application of samples on the PADs: small furrow in which to apply the crushed samples[a]
**PharmaChk (One)**	100%<br>(54.1-100%)	83.3%<br>(35.9-99.6%)	All but one reduced API% samples correctly identified in laboratory evaluation<br>Calibration reference samples run simultaneously with sample being tested<br>Automated sample and calibration handling<br>Photographic instructions	Genuine simulated medicine sample misidentified as failed<br>Degradation of reagents over relatively short time<br>Sample destruction and extraction required<br>Chemicals required<br>Computer required	Capital and provincial laboratories by experienced analysts as alternative to formal HPLC for detecting falsified and substandard medicines, if API range can be extended	To create the library and software: N/A<br>To test a sample: Medium: Needs ability to prepare all sample solutions and follow on computer instructions		Wider range of APIs<br>Development plan to have device preloaded reagent solutions

Device (N° of API tested in the study)	Ability to identify 0% and wrong API content samples (laboratory results)	Ability to identify 50% and 80% API content samples (laboratory results)	Advantages	Limitations	Supply chain location where the device could be useful[a]	Level of training (chemist opinion)	Notes	Suggestions for improvement
**Progeny**<br><br>**(All seven)**	100%<br><br>(92.5-100%)	16.7%<br><br>(6.4-32.8%)	Simple procedure for reference library creation<br><br>Using the Analyse function would avoid selecting wrong library<br><br>Easy to use for end user<br><br>Large number of in-built reference libraries<br><br>Easy interpretation: Results trusted by users (return of the closest match appreciated)<br><br>Analysis through packaging[d]: good performance through medicine packaging (except through glass vial) and replacement packaging<br><br>Computer not needed<br><br>No specific software needed to export data to a computer	Issue to identify one brand of FC ACA (issue with coating suspected)<br><br>No 80% API samples identified as fail in laboratory evaluation<br><br>Poor sensitivity to identify 50% API samples (except ACA samples)<br><br>Reference library creation: Averaging spectra for reference library creation to take into account variability inter-batch or of dosage units from same batches not possible (spectra individually add in the library)<br><br>Errors to select the right reference library using the 'Application' function/False positives using the 'Analyse' function because of similarities of spectra between brands of the same API<br><br>Longest testing time per sample than other non destructive spectrometers except the Truscan RM (users mentioned slowness)<br><br>Heavy weight, large width<br><br>Touchscreen not very responsive increasing the time to record<br><br>Different functions may be confusing for end users<br><br>Tablet holder difficult to use for small tablets<br><br>Daily calibration with chemicals (provided at purchase)	Throughout proximal supply chain for detecting falsified medicines but might be difficult for pharmacy drug inspection	<u>To create the library and software:</u><br><br>Low: A library could be create by any user<br><br><u>To test a sample:</u> Medium: Needs to be able to conduct calibration check and understand how to conduct experiments. Device analyzes spectra.	Slow set-up and long time taken to record sample; Total testing time not different than the Truscan RM<br><br>Self-corrected user errors (selection of wrong library) has been observed in the field<br><br>No protocol was found either in the manual provided at purchase, nor on the website of the manufacturer, on which functions to be used and how to interpret the results for medicine quality screening. We were informed after the study by the manufacturer that the protocols are available on request with an additional cost.<br><br>Barcode reader could not be tested in this study but its use would likely reduce library selection errors by users	Algorithm for detecting reduced API samples<br><br>Reduce the size and weight[a]<br><br>In-device calibration<br><br>Tablet holder adapted for small tablets[a]

Device (N° of API tested in the study)	Ability to identify 0% and wrong API content samples (laboratory results)	Ability to identify 50% and 80% API content samples (laboratory results)	Advantages	Limitations	Supply chain location where the device could be useful[a]	Level of training (chemist opinion)	Notes	Suggestions for improvement
**QDa**	100% (93.3-100%)	100% (91.6-100%)	Ability to quantitate a single or multiple APIs in a formulation in a single experiment<br><br>Measurements are faster than HPLC in a flow injection analysis setup<br><br>Potential to analyse as many ingredients in the formulation as possible in a single experiment<br><br>A reference list of experimental conditions for each API only required. Few limits to what API can be tested	Need to dilute the samples several times to be in the optimal API concentration range to operate effectively.<br><br>Nitrogen gas supply required<br><br>Mechanical complexity that may make user setup, troubleshooting, and repair difficult<br><br>Destroys sample<br><br>Chemicals required<br><br>Not intuitive software	Capital and provincial laboratories by experienced analysts as alternative to formal HPLC for detecting falsified and substandard medicines<br><br>High level screening device for MRAs without a reference laboratory	<u>To create the library and software:</u> N/A<br><br><u>To test a sample:</u> High: User must be able to prepare chemical solution and understand how to dilute samples (including the mathematics behind it). Ideally need to understand how a mass spectrometer work and how to troubleshoot.	Can be coupled to liquid chromatography for enhanced API identification and quantitation by limiting matrix effects from other ingredients in the formulation, not evaluated in this study.	More intuitive software interface for users, including step by step directions at all parts of the experiments.<br><br>Ability to purchase a pre-built flow injection analysis set-up.

Device (N° of API tested in the study)	Ability to identify 0% and wrong API content samples (laboratory results)	Ability to identify 50% and 80% API content samples (laboratory results)	Advantages	Limitations	Supply chain location where the device could be useful[a]	Level of training (chemist opinion)	Notes	Suggestions for improvement
**RDT**<br><br>**(Two)**	100%<br><br>(73.5-100%)	16.7%<br><br>(2.1-48.4%)	Easy to use<br><br>Integrated quality control (control line)<br><br>Electricity not required	Destroys sample and sample preparation needed<br><br>Interpretation can be counterintuitive (lane appearing at test line means sample fails)<br><br>Limited ability to identify substandards<br><br>Two tests (one at low and one at high concentration) to determine the sample as 'no API' or 'API present but lower amount than stated<br><br>API amount undefined<br><br>Colours of tests sometimes not consistent (light pink to red) which can be confusing to users<br><br>Co-formulated ACT cannot totally be characterized<br><br>Short shelf-life<br><br>Chemicals required	Distal supply chain for screening for iv artesunate and DHAPs containing zero API	-To create the library and software (chemist opinion): N/A<br><br>-To test a sample: Low/Medium: Only ability needed to prepare extractions and dilutions. Some difficulty initially learning how to interpret the data because counterintuitive to common immunoassay cartridge tests.	Although one advantage is that the test has a similar operating procedure to malaria rapid diagnosis or pregnancy test, the results can be counter-intuitive and could result in misinterpretation	Reversing the test line system so that a positive line indicates presence of API<br><br>Wider range of APIs<br><br>Ability to test all API of co-formulated medicines<br><br>Longer shelf-life

Device (N° of API tested in the study)	Ability to identify 0% and wrong API content samples (laboratory results)	Ability to identify 50% and 80% API content samples (laboratory results)	Advantages	Limitations	Supply chain location where the device could be useful[a]	Level of training (chemist opinion)	Notes	Suggestions for improvement
**TruScan RM**<br><br>**(All seven)**	100%<br><br>(92.5-100%)	22.2%<br><br>(10.1-39.2%)	Several batches of the same reference sample can be added to the reference library to take into account variability<br><br>Good sensitivity to identify 80% DHAP samples<br><br>Easy to use for end user, step-by-step screen instructions<br><br>Analysis through packaging[d]: good performance through medicine packaging (except through glass vial) and replacement packaging<br><br>Testing time per sample not significantly different as Progeny, but Truscan RM slower than NIR-S-G1<br><br>When sample fails to match the selected reference library spectrum, the whole library of spectra is searched by the device looking for the closest match<br><br>Does not require computer for field use	Reference library creation: averaging spectra to take into account the variability inter-batch or of dosage units from the same batch not possible (spectra individually added in the library)<br><br>Poor sensitivity to identify 50% API samples (except AZITH, DHAP and ART samples)<br><br>Difficulties to scroll down with buttons when looking for the reference library<br><br>Tablet holder not adapted to larger or smaller sized tablets<br><br>User errors because of wrong selection of reference library<br><br>Initial set-up of master computer and software packages difficult, requiring computer skills<br><br>Specific software needed to export data to a computer<br><br>Bothersome to change tablet holder and cone<br><br>Heavy[a]	Throughout proximal supply chain for detecting falsified medicines	<u>-To create the library and software (chemist opinion):</u><br><br>A computer software knowledge is required to create the methods and also to connect the instrument and the computer<br><br><u>-To test a sample:</u><br><br>Medium: Needs to be able to conduct calibration check and understand how to conduct experiments. Device analyzes spectra.	Analysis time[b] faster than Progeny NB: samples with low intensity signal take longer times<br><br>Barcode reader could not be tested in this study but its use would likely reduce library selection errors by users	Search box to look for a specific reference library[a]<br><br>Only one accessory to scan both through and not through packaging<br><br>Simplified initial setup.<br><br>Algorithm for detecting reduced API samples<br><br>Device should be lighter[a]

[a] Medicine inspectors statements; [b] Analysing begins when the process to obtain a result is started, ends when the device returns; [c] Sampling: begins when the inspector starts to use the device (e.g. opens bag containing tablet to begin sampling; touches and starts to use device); [d] Requires specific reference library 'through packaging'; [e] Developers claim that the device has the potential to test liquids after pre-treatment (drying); [f] Clavulanic acid in ACA, dihydroartemisinin in DHAP and trimethoprim in SMTM can't be tested with the PADs; [g] Interpreting and recording: begins when the inspector starts looking at the result, ends when the pen is put down from recording the result on the record sheet. For devices returning results which require interpretation (e.g. PADs, 4500a FTIR), this includes time take to interpret the result. Ends when the process to obtain a result is started (e.g. 'scan' button is pressed; or PAD is put into the solvent) the result
EP, evaluation pharmacy; FC, field collected; N/A, not applicable; *SM, simulated*
