# Supplementary material for: Evaluation of portable devices for medicine quality screening: Lessons learnt, recommendations for implementation, and future priorities
Source: PLoS Med. 2021 Sep 30;18(9):e1003747. doi: 10.1371/journal.pmed.1003747 (PMC8483386; doi:10.1371/journal.pmed.1003747)
Supplement: S3 Table — (PDF) [file pmed.1003747.s003.pdf]

S3 Table. Degree of difficulty to analyse different medicines formulations with the devices included in the study, relative to the analysis of a tablet

These hypothetical classifications assume the API/excipient are not limiting factors in the detection capabilities of the devices.

	Medicine formulation			
**Instrument**	**Capsule**	**Liquid (water based)**	**Powder**	**Creams/Gels**
Minilab	Same	Easier	Easier	Medium
Progeny	Medium	Higher	Same	Higher
TruScan RM	Medium	Higher	Same	Higher
MicroPHAZIR RX	Medium	Higher	Same	Higher
Neospectra 2.5	Medium	Higher	Same	Higher
NIR-S-G1	Medium	Higher	Same	Higher
4500a FTIR	Same	Higher	Easier	Higher
PADs	Same	Medium	Easier	Higher
RDTs	Same	Easier	Easier	Medium
C-Vue	Same	Easier	Easier	Medium
PharmaChk	Same	Easier	Easier	Medium
QDa	Same	Easier	Easier	Medium

S3 Table summarizes the potential differences in difficulty when trying to test medicines formulations other than tablets with each of the devices evaluated. These classifications are based on perceived difficulty of the experiments, and what potential chemical information can be extracted from these types of medicines when compared to tablets. “Easier” means that one to several steps are eliminated because the medicine is in a form that the instrument can immediately analyse and get the same chemical API information as for a tablet. “Same” means the same exact experimental steps would be followed as with a tablet and the user would get the same API chemical information as for a tablet. “Medium” means that additional or a significant change in the experimental steps would need to be taken, such as performing an extraction or destroying the sample to get an equivalent amount of API chemical information as for a tablet. “Higher” means that many additional experimental steps would be required and that getting the same chemical API information as a tablet would be an additional challenge.

Analysing a powder with destructive devices such as the 4500a FTIR would be easier than for tablets because tablets need to be crushed for analysis. The difficulties of analysing powders vs tablets with the non-destructive spectrometers are similar. For destructive devices, capsule analysis would be on the same level of difficulty as for the tablets. The spectrometers would have additional difficulty analysing the capsules if the non-destructive capabilities of these devices were to be maintained. Due to the thickness of the capsules, the non-destructive spectrometers may not be able to interrogate the API and the resulting data may only be of the capsule material itself. Destroying the capsules and analysing the powder inside would potentially enhance the capabilities to discriminate between good and poor quality medicines based on the API(s). If there were any chemical defects of the capsule itself, they could potentially be picked up by the instruments. If the capsule is within good quality specifications and is a spectral barrier to interrogating the internal contents of the medicine, it would not be possible to determine if the medicine was poor quality or not.

For the devices that require the API to be dissolved in solution, analysing liquids would be easier because this would most likely not require an extraction step inherent with solid samples, assuming no interference from the liquid bulk of the medicine in question. Additionally, devices that conduct liquid-based experiments typically require samples that are significantly diluted to be within the operational concentration range of the instrument. The spectrometers would have the most difficulties analysing liquids because the API(s) may not be in high enough concentration to produce a signal that would overcome the signal of the bulk excipient liquid. One way the Raman instruments could be enhanced for liquid analysis is by using a technique known as surface enhanced Raman spectroscopy, a technique where the user adds gold or silver particles in the sample to boost the signal of the API; however, this would require additional protocol and experimental development for the devices evaluated in this study. The PADs might have difficulties analysing liquids. Attempting to add the liquid medicine to the sample application line might be difficult. The PADs developers attempted to test injectable ceftriaxone with encouraging results.

Cream and gels would be the most difficult sample set to analyse with all the devices used in this study. Since creams and gels contain high amounts of oils and other organic compounds that contribute to the medicines thickness’ or viscosity, the devices that require the API to be dissolved in solution may need an additional liquid extraction step or else the devices may be overwhelmed by the signal from the bulk excipient. Spectrometers in particular may be affected by the bulk excipient that may overwhelm the signal of the API(s). Due to the thickness of some creams and gels, it may be possible to apply the sample to the PAD application line, but this assumes that the sample can dissolve when the water passes through the application line during PAD processing.
