# Supplementary material for: Evaluation of portable devices for medicine quality screening: Lessons learnt, recommendations for implementation, and future priorities
Source: PLoS Med. 2021 Sep 30;18(9):e1003747. doi: 10.1371/journal.pmed.1003747 (PMC8483386; doi:10.1371/journal.pmed.1003747)
Supplement: S1 Text — (PDF) [file pmed.1003747.s004.pdf]

S1 Text. Summary of the multi-stakeholders meeting

Overview

The multi-stakeholder meeting was held in Vientiane, Lao PDR on 9th and 10th April 2018 with attendees from seven MRAs representing sections of inspection, quality control laboratory and regulation, from Laos, Thailand, Cambodia, Myanmar, Vietnam, Indonesia and Liberia (one attendee only) along with observers from the World Health Organization from the Lao Country Office, WHO Wester Pacific Regional Office (WPRO), South-East Asia Regional Office (SEARO) and Geneva; the Global Fund Lao country office; the Asian Development Bank; United Nations Development Programme Geneva (UNDP); the Wellcome Trust and the US Pharmacopeial Convention (USP). Additional staff from the Lao MRA, including from provinces, and from the Lao University of Health Sciences (UHS) also attended the meeting.

On the first day of the meeting, after the meeting was opened by Dr Somthavy Changvisommith – Director of the Lao Food and Drug Department, who welcomed the participants, Dr Klara Tisocki of WHO-SEARO discussed the importance of quality medicines for public health and the importance of screening devices to empower key actors throughout the pharmaceutical supply chain. She raised questions that urgently need to be answered such as how the screening devices can fit into the regulatory activities of MRAs.

Dr Céline Caillet of LOMWRU/IDDO then presented an overview of the study and of the devices included in the different phases of the project, explaining the basis of the technologies studied. The main findings of the evaluation of portable devices from the current project were then presented by Dr Serena Vickers, of LOMWRU/IDDO, and Stephen Zambrzycki of the Georgia Institute of Technology. The participants were given opportunities to handle and use six of the devices included in the field evaluation (4500a FTIR, MicroPHAZIR RX, NIR-S-G1, PADs, Progeny and TruScan RM) with explanation by the LOMWRU/IDDO team. This formed a framework for the discussions on the optimal use of devices by MRAs with the aim to facilitate intra- and inter-country discussions. Mr Lukas Roth of USP gave an account of the parallel USP project on medicine quality screening devices.

On the second day, the cost-effectiveness analysis results were presented by Dr Nantasit Luangasanatip and Professor Yoel Lubell of MORU. Three hours of country group discussions were then held, facilitated by the WHO representatives with suggestions of points to discuss developed by the study team. Mr Lukas Roth of USP then summarized the country group discussions and a final discussion, with all MRAs representatives and observers together, was held.

Summary of discussions on the devices

Minilab

The Minilab, that is widely available to MRAs in the participating countries, was mentioned as an important device in practice. Indeed, it was described as able to provide interesting data on a sample quality because of its ability to assess whether the API is present or not, whereas the spectrometers presented at the meeting provide information on the whole formulation only. However, major difficulties of sourcing and the unaffordable costs associated with procurement of reference standards, consumables and TLC plates for Minilab were mentioned by most of the regulators of the countries where the Minilab is (or was) in use. In addition, as far as we are aware the Minilab is not used at the point of sale by medicine inspectors in these countries, but rather in an office or laboratory by trained technicians.

Spectrometers

Although most of the spectrometers were viewed as easy-to-use, and less time consuming than other technologies discussed, frequently mentioned issues for implementation of spectrometers in PMS were their high costs, the need for the creation of reference libraries, and requirements for calibration and performance verification.

Overall the Raman devices tended to be preferred by the MRAs present over the other devices. In several countries the TruScan was already in use by regulatory authorities or the police at the time of the discussion, which may have played a role in this preference towards the TruScan. One advantage of the Progeny over the TruScan that was quoted, was that the Progeny did not require a specific software to export data to a computer.

The NIR-S-G1 was perceived as the easiest to use, with smartphone capabilities that were much appreciated by most meeting participants. However, rather paradoxically, regulators from several countries agreed that its small size and less robust aspect as compared to other devices, made the NIR-S-G1 appear less reliable than more costly devices. In addition, the lack of calibration function by the user and of performance quality checks (see paragraph below) with the version evaluated in this study (according to the developer the newer version will have a calibration check) were perceived as barriers to reliable use.

One regulator perceived the 4500a FTIR as especially reliable, with the major factor being the visual appearance of the device. This regulator, who also had quality control laboratory experience, mentioned that analysing the powdered tablets yields more reliable results than testing tablets intact, because the ‘core’ of the tablet is tested, thus avoiding interfering signals from any coatings.

<u>Costs</u>

The very limited MRA budget allocated to PMS was mentioned as a barrier to implementing screening technologies by the different country regulators. Calibration, maintenance (cost of battery replacement for example) and performance quality checks associated costs were recurring concerns raised by regulators towards implementation of the spectrometers in their environments.

Regional procurement strategies to purchase substantial numbers of units of high cost devices from one manufacturer might significantly reduce the capital equipment costs.

Reference libraries

The costs and logistical considerations associated with the creation of libraries were of concern, given the large number of brands available on the market. Some regulators especially mentioned concerns regarding the costs and time associated with making sure that the reference library samples are of good quality.

There were differences of opinion regarding which entity could be responsible for creating the reference libraries among the different regulators. For some MRAs, the regulatory agency was perceived as the key actor to create reference libraries because of the privileged ‘relationship’ with manufacturers and procurement agencies. Indeed, some regulators believed that the provision of different batches of genuine samples by the manufacturers at the time of registration should be a requirement for marketing authorization. If any minor or major changes of formulation was to be made, the manufacturer should apply for new registration approval.

In some countries one batch of all brands submitted by manufacturers for registration has to be tested by compendial testing before marketing authorization approval is given. This batch could be used for reference library creation but only one batch will not take into account inter-batch variability.

Other participants suggested having one organization/institution, in a regional approach, to create and update reference libraries using reference medicines obtained from manufacturers directly or by the MRAs.

Difficulties in collecting ‘genuine’ but unregistered medicines (highly prevalent in some countries according to regulators) that, *‘have not undergone evaluation and/or approval by the National or Regional Regulatory Authority (NRRA) for the market in which they are marketed/distributed or used’*[1] were stressed as a barrier to the creation of reference libraries. Minilab was thus viewed as a more useful tool in this context due to its API-specific approach, the provision of reference standards on purchase, the lack of ‘matrix effects’ of the excipients on the

[1] SF Medical Products Group, Essential Medicines and Health Products WHO. WHO Member State Mechanism on Substandard/Spurious/Falsely-Labelled/Falsified/Counterfeit (SSFFC) Medical Products. In: Seventieth World Health Assembly [Internet]. Geneva, Switzerland; 2017. p. A70/23: 33-36. Available from: http://www.who.int/medicines/regulation/ssffc/A70_23-en1.pdf?ua=1

result (compared to spectrometers) and the fact that new APIs are being added regularly to the Minilab system, allowing for a broader spectrum of screening.

If the country medicine regulatory agencies were to implement spectrometers in PMS, an incremental roll out of reference libraries, starting with several brands prioritized on a risk-based approach, was also suggested as the way forward.

Calibration and performance verification

Regulators were concerned about the process of the calibration, quality control of performance and the maintenance of the devices such as the expected lifetime of batteries, and the associated costs. These may be a barrier for the sustainability of the devices use. They regarded some of the costs to replace batteries as prohibitive in their settings.

Concerns about the NIR-S-G1 for which no calibration was available for the version of the device used in our study, were raised as a potential barrier for ensuring performance quality of the device. According to the developer, the latest version of the device (not evaluated in this study) contains a calibration check to ensure the device is operating within optimal operation conditions; the user will scan a piece of plastic and if the device result is out of specifications, it must be sent back to the manufacturer for repair.

Paper analytical devices

According to the Lao BFDI medicine inspectors who participated in the field evaluation of the project, they felt that the results produced by the PADs are too operator dependent - ***'Each person has a detection limit'***- and were not in favour of using the PADs. When mention of a PADs smartphone reader was made, some still felt that a camera might not give accurate results whilst others believed it would help result interpretation. Evidence as to the PADs smartphone reader performance accuracy are required. Issues with the stability of the PADs under tropical conditions was raised as a potential barrier for their use.

Supply chain level

Spectrometers were favoured for their use in the field, at the retailer/outlet level and at the borders/customs by the regulators, except the 4500a FTIR that was mentioned as potentially useful at checkpoints or in a laboratory setting. This device was also perceived as interesting for raw material analysis. The PADs were perceived by some regulators as potentially useful in a laboratory or at border checkpoints or, for remote health workers (e.g. village health workers) who could incorporate them into their work on pre-existing disease programs. Their cost was perceived as low compared to other devices, but still high when considering that it is a single-use device, and that it is limited to testing only some APIs.

Post-marketing surveillance strategy

With the current state of knowledge about the devices presented during the meeting, it seemed likely that more than one technology should be used in PMS. Multi-level testing with different technologies was suggested as the best option. For example, at border checkpoints, a screening technology that gives a fast result, operated by staff without a high level of training and no or little user interpretation (e.g. a spectrometer) might be preferable. From that screening, samples could be submitted for secondary analysis with the Minilab or PADs, for example. Finally, a subset of samples could be sent for confirmatory compendial testing.

When asked about their choice of strategy as to whether to send a sample for confirmatory testing if the test with a device results in a 'fail' in the field, regulators felt that retesting the failing samples at least once would be a good option. However, the need for more data on device performances are required to refine the strategies that are perceived as device-dependent.

Acting upon suspicious medicines - strengthening regulatory systems

Spectrometers were perceived by some regulators as a great benefit for public health because it would give immediate results to detect falsified medicines, which would reduce the time to take action. There seemed to be a common agreement that implementing screening technologies in PMS should be part of a wider system that is highly setting dependent. Some regulators mentioned that in their countries there is currently no law to implement regulatory action when a medicine fails a screening technology. The regulators need to wait for the confirmatory analysis (it can take up to several weeks). On the other hand, it was mentioned that some countries where Raman spectrometers are currently in use, adopted an approach that medicines failing the device tests are put in quarantine until the confirmatory analysis is done.

Gaps of evidence

Spectrometers

The lack of evidence on the ability of the spectrometers to identify substandard medicines was the main concern of regulators, as most mentioned the substantial problem of substandard medicines in their countries. Knowing the limit of detection of API content by the spectrometers used for API quantitation would be of great interest.

The limit of detection in terms of API amount relatively to the weight of the whole formulation was also mentioned (e.g. for levothyroxine formulations containing only micrograms of API).

Uncertainties about the abilities of the devices to accurately test coated tablets, liquid formulations, capsules and creams/gels were mentioned as major gaps in the evidence. In addition, the performances of the devices to test through packaging should be more widely investigated.

A recurring gap addressed during the discussion was whether the spectrometers were able to accurately identify poor quality fixed-drug combinations with multiple APIs such as anti-tuberculosis medicines containing four co-formulated APIs. Minilab was viewed as a useful tool

in this context due to its API-specific approach without the need for a lot of additional work as could be needed for spectrometers. Multivitamin tablets quality was mentioned as a major issue in one participating country, where they cannot currently be tested with the equipment available in the national quality control laboratory.

The memory capacity of the devices, in terms of the number of reference libraries that can be saved, in addition to the number of samples that can be tested was raised by several participants in the meeting. These data were thus added to the second article of the PLoS NTD's series.

Worries about the level of knowledge/training required to set-up instruments were raised.

Other questions were asked about the possibility to use the same reference libraries in different technologies; the number of batches needed to make a good reference library; the device performances in different climates; how the acceptance threshold for quality in spectrometer algorithms is determined and validated (e.g. for the 4500a FTIR).

Some regulators also enquired about the differences of spectra between different brands of the same API/combination of APIs with spectrometers, thinking about it as a way to reduce the number of genuine reference samples needed to create reference libraries.

Other comments

Other gaps of evidence underlined by regulators were the potential abilities of devices to detect degraded medicine and medicines with poor dissolution.

Some regulators acknowledged that it would be of great interest to know whether any of the devices discussed are already in use in any country for routine drug inspection, to build upon experience from other countries.
