# Supplementary material for: Evaluation of portable devices for medicine quality screening: Lessons learnt, recommendations for implementation, and future priorities
Source: PLoS Med. 2021 Sep 30;18(9):e1003747. doi: 10.1371/journal.pmed.1003747 (PMC8483386; doi:10.1371/journal.pmed.1003747)
Supplement: S1 Fig — Extract from the USP technology review reports of 3 devices: CBEx (Raman), ASD QualitySpec (NIR), and Target-ID (FTIR) (see https://www.usp.org/global-public-health/technology-review-program). Basic user: a user with the ability to follow a standard operating procedure or work instruction to set up and run the instrument and collect data. Intermediate user: a user with the ability to develop and modify methods and evaluate and interpret results. Advanced user: a user with the ability to train other staff and perform basic troubleshooting. Nontechnical experience: a trainee with no prior laboratory experience and no background in one of the physical sciences (e.g., chemistry). Technical experience: a trainee with prior experience working in a laboratory and/or a background in one of the physical sciences. Specialised experience: a trainee with theoretical and practical experience using the technology or the technique underpinning the technology. FTIR, Fourier transform infrared; NIR, near-infrared. (PDF) [file pmed.1003747.s005.pdf]

User Experience	User Type		
	Basic	***Intermediate***	***Advanced***
Non-technical	1 to 2 days	1 week	2 weeks
Technical	1 day	1 day to 1 week	1 to 2 weeks
Specialized	1 to 2 hours	1 day	Less than 1 week
